# Observations on Expansibility as a Vital Property, and on the Influence of the Capillary Tissue, over the Circulation of the Blood

**Published:** 1828-08

**Authors:** 


### REVIEWS AND BIBLIOGRAPHICAL NOTICES.

---

Art. IV. *Observations on Expansibility as a Vital Property, and on the Influence of the Capillary Tissue over the Circulation of the Blood.* By Hugh L. Hodge, M. D. *Lecturer on the Principles of Surgery in the Philadelphia Medical Institute.* [North American Medical and Surgical Journal, for July, 1828.—pp. 37.]

Notwithstanding 200 years have elapsed since the discovery of the circulation of the blood, that great and sustaining function of the animal system, is far from being thoroughly understood. We know, with the immortal Harvey, that, in man and the other mammalia, the blood is projected from the left heart, into a common conduit, which ramifies indefinitely; that it is received into the veins and returned by two trunks to the right heart, whence it is thrown into another arterial trunk, and again received from its ramifications by four veins, and restored to the cavities from which it first issued—having completed the tour of the whole body. Of the structure of the hydraulic apparatus, by which this *unique* circulation is effected, we know something—indeed, a great deal—but we do not know the whole. The *cardiac* portion consists of parts which can be seen and examined, but the *capillary*, are minute, intangible, in a great degree invisible, and constitute, in many of their parts, a kind of *terra incognita.* Having but a partial acquaintance with the anatomy of the circulation, it is not wonderful, that our knowledge of its physiology, should be imperfect;—for a machine must be surveyed in all its parts, before its mechanism can be understood. But the *modus operandi* of the central parts, themselves, is not yet fully revealed—notwithstanding the descriptive anatomy of those parts has, for ages, presented an aspect of great perfection. For example, it is still uncertain in what manner the cavities of the heart are dilated: and how far that organ performs the office of a sunction pump, upon the columns of blood in the great veins; or how its action, united with that of the larger arteries, occasions their pulsation. If doubts rest upon the physiology of that structure, which is well understood, the functions of that which is nearly unknown, must be enveloped in obscurity. Hence, there have been many "vain imaginings," as to the manner in which the small vessels receive, transmit, and vary the distribution of the blood. Of the opinions, which may be regarded as deductions from fact, there are two, which divide the physiological world. First, that the capillary vessels have a constant contraction, which tends to diminish their diameters and to resist the action of the heart; which, however, possesses such relative force, that the lateral contraction of the capillaries operates, of necessity, to push the blood forward into the veins. Secondly, that the capillaries, like the heart itself, alternately dilate and contract; thus receiving blood from the larger trunks by a suction, which co-operates with the *vis a tergo* impressed by the heart, and sending it forward into the veins. Either hypothesis may, perhaps, explain the phenomena of the circulation, when it is *in equilibrio*, but such a state is rather imaginary than real; for a perfectly equable distribution of the blood throughout all the organs, that is, a condition in which all the vessels are, simultaneously, filled to the same degree, in proportion to their capacities, does not, in fact, ever exist. The vital fluid is never equally distributed; but is found accumulated, successively, in all the organs and tissues of the body, according to the excited state of their functions. Now, no theory of the capillary circulation, that will not explain these physiological and pathological determinations into the different organs, and the consequent disruptions of the balance of the circulation, should be regarded as true or useful. To explain them by a reference to the actions of the heart is impossible; seeing that the blood is thrown by that engine into a common trunk; and, therefore, that the capillaries are all fed from the same fountain, and, of course, affected in the same manner, by the changes which it undergoes. To the capillaries, themselves, must we look for, the power which draws the blood into one organ, and withdraws it from others. But, on the first hypothesis, how can they do this? They do it, we are told, by doing nothing; that is, by contracting with less energy, in a particular organ, whereby, that organ suffers accumulation, from the general pressure of the vascular system; which directs the blood towards the weakest part—just as in any system of elastic tubes filled with a fluid, the one which is weakened will of necessity become distended. Every case of local plethora has, then, for its proximate cause, reduced power and contraction of the capillary vessels, in which it occurs. Now, this plethora is of two kinds, that which occurs in organs, when acted on by their specific stimuli, and thrown into the orgasm necessary to the performance of their proper functions; and that which results from abnormal irritation and constitutes disease. One of these, however, is natural and healthy, the other unnatural and morbid; and to refer them to the same condition of the same function—that of contraction—is to violate one of the soundest rules of philosophical logic. Moreover, it seems incredible, that the proximate cause of the high excitement which organs exhibit, when performing their functions, should be a state of debility.

We have, therefore, long been disposed to prefer the theory, which assigns to the capillaries an active agency in disturbing the equilibrium of the circulation; and in attracting increased quantities of blood, into particular parts; and have even ventured, when attached to the medical department of Transylvania University, to adopt and inculcate the opinion, that they possess dilatability, as well as contractility. To support this theory, is the design of the essay of which we now proceed to speak.

In the following paragraph, our author sets forth the object of his inquiry:

"All physiologists agree, that capillaries have the vital property of contractility, that they contract on the application of certain stimuli, and that the result of this contraction is the expulsion of their contents. The object of this paper, however, is to show, that capillaries have a vital property of expansibility,—that they dilate under the influence of certain stimuli, and that the effect of their dilatation is to draw blood into their cavities from the supplying arteries, and thus materially to facilitate the motion of this fluid in the large as well as in the small vessels. The facts to be presented in support of this opinion, will render it at least probable, that expansibility is a property, not only of the capillary tissue intervening between arteries and veins, but of all capillary vessels,—of the radicals of the veins, lacteals, lymphatics, and ducts of glands, and that it is possessed in a slight degree by the heart and arteries, also, as well as by the tissues confessedly erectile, as the penis, nipple, iris, &c." p. 3.

In the succeeding paragraph, he reasons with those who think, or suppose they think, that it is more difficult to form an idea of expansion than of contraction, in living tubes; and declares, with justness, that the latter is, in fact, as remarkable as the former. He then tells us that expansibility, as a vital property, is not a new idea, but was held by Bordeu and Hunter, and has lately found many advocates in Europe, and the United States. His object seems to have been, to embody all the known facts in support of that opinion, and these he has arranged under the following heads:

"The points to be discussed will all be embraced in the three following propositions, which I shall endeavor to sustain by facts and arguments.

"I. The capillary and arterial tissues do not dilate passively from a vis a tergo, derived from the heart and arteries.

"II. Some tissues possess expansibility as a vital property; that is, are capable of expanding under the influence of certain stimuli. This property is possessed most remarkably by the capillary system intervening between the arteries and veins, and by the radicles of veins, of the absorbents, whether lacteal or lymphatic, and of the excretory ducts of glands. That it also exists in the heart and arteries, perhaps also in the veins and the ducts of glands, as well in those structures denominated erectile, as the iris, penis, nipple, &c.

"III. The circulation of the blood in the arteries is powerfully aided by this dilating power of the capillaries, drawing the blood into their cavities; and this power of the capillaries is the almost exclusive agent in all increased local determinations of blood.

"IV. During the activity of inflammation, an increased quantity of blood flows through the affected part." p. 5.

In support of the first proposition, he cites many acknowledged and familiar facts; of which we consider it unnecessary to extract more than a single group:

"Contusions, when so severe as to paralyze the vessels without destroying their vitality, leave a part pale for a longer or shorter period according to the violence of the blow. Boyer, in his Surgery (tome i. p. 105), notices several cases of local asphyxia from blows, "in which the heat, the sensibility, the movements, the pulsations of the arteries, in fine, all the phenomena of life, after having been apparently annihilated for many days, gradually returned and resumed all their former vigour." He cites from De La Motte the case of a boy whose right hand was completely paralysed by a blow with a billiard stick, received on the outside of the forearm; "the hand, for ten days, appeared to be dead; but at the end of that time the heat gradually returned, the beating of the arteries became perceptible, and the hand was restored to its natural condition." How can the supposition of passive dilatation be consistent with these facts? The vis a tergo from the heart and arteries, was perfect; other parts of the capillary system are dilated; but those vessels, whose powers are most exhausted, instead of being most dilated as they would be, if the theory were true, are the most constricted." p. 6.

The second proposition, that *some tissues possess expansibility as a vital property; that is, are capable of expanding under the influence of certain stimuli*, has received a great share of our author's attention; and is sustained by an array of facts, too well ascertained to be rejected, and too pertinent to be easily explained away. He begins, by referring to the acknowledged existence of erectile tissues, as first pointed out by Bordeu, and offers reasons for ascribing the 'expansile power to the vascular system.' We believe with him, that it belongs to that system, and resides in the nascent veins as well as ultimate arteries. We believe still further, that it is universal, in other words, belongs to *all* the minute vessels, but is greater in some than others; being proportionate to the physical necessity which may exist in different organs for copious, occasional supplies of blood.

In the first place, our author considers this property as appertaining to the absorbents, without which, he thinks, we cannot explain the function of absorption.

Secondly, he treats of it as a quality of the sanguineous capillaries; and the whole section is worthy of an attentive perusal; but we must limit ourselves to the following extracts:

"Certain stimuli or irritants are immediately followed by an enlargement of the capillaries; there is no evidence of prior contraction and subsequent exhaustion.

"Caloric is a universal stimulus. When a portion of the cutaneous tissue is immersed in warm air or warm water, an immediate distention of the capillary vessels is observed. This dilatation by the stimulus of heat is as immediate as the contraction of a muscular fibre from any mechanical irritant; the one is as direct an effect as the other. There is no evidence of exhaustion or debility of the capillary vessels; for all the phenomena peculiar to life are augmented, such as heat, scarlet redness, circulation, and secretion. Neither should the effect be attributed to an increase of the vis a tergo; for, 1st, The enlargement of the capillaries occurs before the excitement of the supplying arteries; 2d, It is often great when there is no evidence of increased action of the larger vessels; and 3d, Such dilatations may often be excited by caloric, when the actions of the heart and arteries, including the supplying arteries, are exceedingly depressed. In all cases of prostration, where the pulsations of the heart and arteries can scarcely be perceived, where the surface is cold and pale, and all the organic vital phenomena are greatly diminished, warm applications are always resorted to; and when they redden the skin and produce inflammation or vesication they are advantageous. Where, in such cases, is the vis a tergo to dilate the capillaries? or what reason is there for imagining that the effect of the stimulus on these pale, bloodless, and weakened vessels, is to contract them, and thus to exhaust their power that they might yield to a vis a tergo? Further exhaustion would be death, which caloric, as every practitionerknows,would rather prevent than accelerate.

"These remarks respecting caloric may be extended to most other irritants. The puncture of a needle or the sting of a wasp, is immediately followed by redness; a mote under the eyelids instantaneously causes an enlargement of the vessels of the conjunctiva; a drop of nitric acid reddens the skin, and so of other irritants. In none of these cases is there evidence of contraction preceding dilatation; or of exhaustion directly or indirectly produced.

"Internal stimuli often enlarge the capillaries without any evidence of an increased vis a tergo. How instantaneous is the appearance of a blush, mantling the face and neck from the operation of certain passions. The enlargement of the small vessels, in blushing, is the result of a nervous irritation, not of increased action of the heart; for, 1st, This viscus is often undisturbed: 2d, In all instances the blush is partial; if owing to the contraction of the heart it would be universal: 3d, in violent palpitations and other increased actions of the heart, the capillaries are not distended as in blushing, but are often remarkably contracted. Their enlargement, as well as their contraction, must depend on inherent powers.

"The remedial agents, which increase the diameters, as well as the functions of capillaries, without augmenting the action of the heart and arteries, in other words, the vis a tergo, are very numerous. To this head must be referred most of the diaphoretics, diuretics, expectorants, and cathartics. Mercury also will often produce local dilatations, secretions, and even inflammations of the skin, gums, tongue, and salivary glands, without any evident excitement of the general circulation. In such cases, is not the hypothesis unfounded, which maintains that the vessels, thus acted on by internal stimuli, and having all their vital phenomena increased, must be debilitated before they enlarge?

"Even the dilatations of capillaries from the diffusible stimuli, wine, brandy, &c. which do disturb the heart and arteries, cannot be owing to a mere mechanical impulse; for, as already hinted, the enlargement is often in an inverse ratio to the vis a tergo. Thus, when the action of the heart is violent, the capillaries are often undistended; and their distention is often very great when the cardiac pulsations are languid.

"Again: Whenever any excitement is manifested in the functions of a tissue, organ, or apparatus; or when any new or increased demand is made on such tissues or organs, the capillary vessels dilate to meet the emergency.

"Compare the different conditions of the capillary system in childhood and old age. In the former, all is activity, not only to replenish the various secretions and excretions so abundant in infancy, but especially to furnish the materials for growth. The capillary system is full and tense. In old age, the phenomena of life are diminished, nutrition and secretion are lessened; while wrinkles and emaciation are substituted for the fullness and tension of a prior stage of existence. The same difference is observed in the peripheral system of infants when in health and when diseased; and of the extremities before and after the occurrence of paralysis. Where the phenomena of life are most displayed, the capillaries are most developed; where they are diminished, these vital tubes are proportionally smaller. Surely, in the former case, they are not debilitated and passively dilated.

"The same remarks are applicable to all *general* excitements of the capillary system. Are not the vital energies in greater exercise, and the capillaries, at the same time, more dilated in an inflammatory fever than in a typhous fever, or in hectic wastings; during the ruddy excitement of health than during syncope; in the hot than in the cold stage of fever; during the stage of reaction than in that of collapse from concussion of the brain; when under the influence of heat than of cold; while the individual is burning with anger than when torpid with fear? That the capillaries in these and similar cases are not distended by an increased energy of the heart was proved in our first proposition.

"The same enlargement of the capillaries is observed in all cases of *local* excitement: cases in which no one contends that there is a diminution of any of the vital phenomena. The capillary vessels are enlarged in the growth of tumors; in tissues or glands for their increased secretion, whether temporary or continued; in the heads of animals which are renewing their horns; in the organs of generation of those female animals which are in season; in the brain and face, during excitement from moral or physical causes; in the uterus after conception, and even before the descent of the ovum; in the iris, nipple, and penis when appropriately excited. In none of these cases is there any evidence of exhaustion of power or an increased vis a tergo. In all, the manifestations of vital energy are increased.—pp. 14.

"During the acute stages of inflammation, the parts are very tense and hard; and moreover, when such parts are covered by any unyielding membrane, as hardened cuticle, fascia, periosteum, &c, the phenomena of inflammation are greatly enhanced, and the progress of the disease to suppuration or gangrene is hastened, while the fascia or membrane becomes very tense. Whence the distending power in such cases? Is it intrinsic or extrinsic?–Not extrinsic, because 1st, The distention is not proportioned to the excitement of the heart and arteries; being often great when this excitement is at the healthy standard, and sometimes even when the general circulation is greatly depressed. 2d, If the swelling and tension were owing to a vis a tergo, the resistance of the membranes, so far from aggravating, ought to cure the inflammation by restoring the equilibrium between the resistance of the small vessels and the vis a tergo. The passive distension of the former would be effectually resisted and the balance restored, which, according to the seductive simplicity of the *mechanical hypothesis* of Dr Philip, is the only desideratum in the treatment of inflammation. Hence Dr Lucas, one of his disciples, recommends pressure as one of the most powerful remedies for this disease; but every practical man knows the mischief produced by pressure from bandages, ligatures, tendinous sheaths, fasciæ, &c. during the acute stages of the phlegmasiæ; for, 3d, We find that the removal of pressure moderates the symptoms, shortens the disease, and facilitates resolution. Hence one advantage derived from fomentations and poultices in superficial inflammations in softening the indurated cuticle; hence the necessity for dividing in a transverse direction the tendinous sheath in paronychia and the fibres of faciæ during sub-aponeurotic inflammations. Relief could not be afforded by such measures, if the dilatation were passive; for the vis a tergo being increased and the capillaries being debilitated, the distension would continue augmenting until rupture or mortification should ensue.

"This objection alone, I regard as sufficient to overthrow the whole hypothesis of our opponents. When inflammation disappears, they say the balance is restored between the capillaries and the supplying vessels,—the disproportion between the resistance and the vis a tergo having ceased; but how can this ever occur spontaneously, that is, without the interference of the surgeon. The more the palsied capillaries are distended, the more incapable are they rendered of resisting the impetus from the supplying vessels. How then is the supposed necessary balance restored? How can the capillaries recover sufficient energy to resist an impetus, which, on their supposition is continually increasing? Rupture or gangrene we should imagine must be the result of every inflammation, in which art does not interfere with its pressure or its astringents.

"The distending power not being therefore extrinsic, depending on a vis a tergo, is *intrinsic*, and its exercise is the result of irritation. For experience shows that when the irritation is removed, the tension disappears: the same capillaries exposed to the same increased vis a tergo, will often diminish in size on the removal of the cause. Remove a mote from the conjunctiva, or a splinter from the skin, and the enlarged vessels are lessened, and the inflammation is resolved, notwithstanding the excited condition of the supplying vessels. On the contrary, the increase of irritation is necessarily followed by a more violent inflammation; its phenomena becoming more intense in proportion to the severity of the irritant, until the vital powers of the part become exhausted and gangrene ensues. Irritation, therefore, and not the vis a tergo, is the cause of the tension of inflamed parts. This irritation is generally increased by pressure, which hence aggravates the phenomena and accelerates the progress of inflammatory affections.—pp. 16—17.

In the third place, he enquires into the expansile power of the larger arteries, which he believes to have an existence, but in a less degree proportionably, than in the capillaries. In proof of this branch of the subject, he relies chiefly on pathological facts, most of which are familiar to the profession. We shall, therefore, pass over them, to lay before our readers, his comments on the experiments of Dr. Parry and Mr. Hastings, whose works on the Pulse, and on Bronchitis, are but little known in this country.

"Numerous experiments upon blood vessels made by distinguished physiologists, with other objects in view, prove the expansile power of arteries. I shall quote facts from two authors only, each of whom maintains opinions opposed to those which I am advocating. Mr Caleb H. Parry,* in his 23d experiment, after having exposed and carefully measured both carotid arteries, tied one vessel: the other increased in size. When this was also tied, it resumed its original dimensions; proving that the previous distention was not owing to a vis a tergo. To this conclusion, Parry himself was led; for he maintains as the result of numerous experiments, that the arteries are always during health distended beyond the size to which

*Parry on the Pulse, Ed. 1816.

their elasticity would bring them (p. 69); and moreover, that this forced distension of an artery does not depend on the impulse of the blood from the left ventricle, (p. 71). This distension, he observes, is increased in local determinations of blood; and as it does not depend on the elasticity of the arteries, nor on the systole of the heart, "we are compelled to admit as the cause of the common dilatation of the larger arteries, and still more forcibly of that which is preternatural, the mechanical distending power of the blood which they contain."—p. 78. This explanation, however, is unsupported by facts; and it would be difficult to conceive how the blood, when in rapid motion through the arteries, could mechanically dilate one vessel and no other, unless some change previously occurred in the parietes of the tube.

"The second author to whom I refer for facts, is Mr Hastings,* who, after many and diversified trials, asserts that mechanical and chemical irritants applied to the larger arteries will often excite contractions and dilatation of the vessels in the part irritated. "This increase of motion did not arise from any modification of the heart's action; for the degree of dilatation and contraction in the unirritated part of the vessel nearer the heart was unaltered"—p. 26. Neither is the dilatation owing, as Mr Hastings would seem to intimate at page 31, to a diminution of irritability or to a suspension of power, for contraction would then be suspended instead of being augmented; nor to relaxation after prior contraction, for such alternate relaxations and contractions do not occur in muscular fibres (those of the heart alone excepted, to which we shall presently allude) when mechanically or chemically irritated. A tonic contraction exists, so long as the irritant is operative. The increased pulsation of arteries under the direct influence of stimuli, is, therefore, but a more clear manifestations of their normal actions;—dilatation being the result of stimulation as evidently as contraction. This is a very fruitful subject. It embraces all questions relative to the causes, nature, and varieties of the pulse, and admits of numerous illustrations. But the facts already adduced, taken in conjunction with the well known varying condition of the pulse in cases of sedation or diminished excitement of parts, as well as in those of irritation, prove that its condition, as to size, tension, and strength, does not depend solely on the systole of the left ventricle, but, at least to an equal extent, on the degree of excitement in the arteries; and that the actions of the arteries are greatly influenced sympathetically by the ever-varying conditions of the capillary tissue to which they are distributed. The formation of the pulse depends on the heart; but its size and tension, on the degree of excitement existing in the arteries and capillaries, so long as there is a free supply of blood.—pp. 20—22.

*On Bronchitis, London, 1820, p. 24, et seq.

Lastly, he comes to examine into the expansibility of the heart, which he believes to be real; and shows that many physiologists have agreed in its *existence*, while they disagreed as to the *mechanism* by which its dilatation is effected. Without citing proofs of that which is so unanimously admitted, we shall conclude our notice of the second proposition, by quoting his final remark.

"The heart would hence appear to possess an active dilating power, exercising a slight suction influence over the blood in the large veins near this organ, and thus facilitating, instead of opposing the ingress of the blood. Evidence, I think, is yet wanting to prove, that any part of the circulatory apparatus, during its healthy condition, resists the motion of the blood. Each portion co-operates, and beautifully harmonizes with every other portion, in carrying the vital fluid through the circulation,—an idea to be further illustrated in the ensuing proposition.—p. 23.

His third proposition is, that 'the circulation of the blood in the arteries is powerfully aided by this dilating power of the capillaries, drawing the blood into their cavities; and this power of the capillaries is the almost exclusive agent in all increased local determinations of blood.'

In verification of the two members of this proposition, he has accumulated a great body of facts; though as he justly remarks, if the preceding propositions are true, this must flow from them as a corollary. In the first place, he surveys the circulation of the sap in vegetables, and finds abundant evidence, as he thinks, of the existence in them of a power of suction, dependent on the expansibility of their vessels. We have room to extract but a single paragraph:

"The sap ascends when the trunk and branches are subjected to the influence of a warm atmosphere; but it remains in the root when such stimulus is not applied. It is manifest, that the caloric, in such cases, operates on the superior or exposed portions of the plant; the roots, buried in the soil, being comparatively unaffected by atmospheric variations of temperature. Examination proves, that parts thus stimulated swell, and, from a comparatively dry state, become moist and suculent; in other words, that the vessels dilate under irritation, attract or suck in the nutritious fluids, whence result leaves, buds, blossoms and fruit. There is no vis a tergo to distend the whole peripheral system of vessels, and in such textures, dilatation must precede the entrance of fluids.—p. 24.

Passing from plants to animals, he presents us with various appropriate observations, of which we shall transcribe the following:

"Dr Philip and Dr Hastings* state, that in live dissections, the circulation in the smaller vessels will continue for several minutes after excission of the heart. In the mesentery, Dr Philip has observed that the circulation proceeded with regularity for half an hour after the removal of the heart†. If contraction were the only vital action, the blood would be driven out of the small vessels; and as no vis a tergo existed from the heart, their dilatation could not occur, but the vessels would remain collapsed and the part bloodless, as in syncope and local asphyxia. Professor Horner also informs us, that during live dissections, the capillary vessels remain distended, after the heart has ceased to act, and so long as any blood remains in the arteries,—the larger vessels becoming first empty, then the smaller ones, and finally the capillary tissue.

"These phenomena cannot depend on mere vital contraction of the vessels: for such contraction would occur more readily in the capillary system, the irritability of which is greater than that of the arteries; and this contraction would not, as shown in Proposition I., be overcome by the vis a tergo from the contraction of the arteries. Hence, the influence from the heart being removed and the capillaries contracting, the blood would remain in the arteries, on the supposition that contractility were the only vital property. But the arteries are found empty before the circulation has entirely ceased in the capillaries, and while these last remain turgid. The capillaries hence manifest a dilating power, drawing blood into their cavities, and assisting the arteries in discharging their contents. This being effected, the contractile powers of the capillaries throw the blood eventually into the venous system where it remains. In this way, I think, the emptiness of the arteries after death may be satisfactorily explained.

The above explanation is confirmed by attending to those few cases in which the arterial system remains distended: as when death has been suddenly caused by some powerful impression, as by lightning, a blow on the stomach, &c. Here the whole of the circulatory apparatus appears to be paralysed, and the blood is generally found fluid. No vascular action continuing after the cessation of the vis a tergo from the heart, the blood remains at rest.

Stimuli cause an afflux of fluids to the part irritated. In Proposition II. *g*, *h*, *i*, *k*, various examples were detailed to prove the

*Essay on Bronchitis, p. 51.

†Essay on Vital Fuuctions, 2d edition.

active enlargement of the vessels under irritations, in all such cases, fluids flow in increased quantities to the part. Whence this afflux? It is not owing to a vis a tergo from the heart and arteries, for this remains as before, and the capillaries are not debilitated (Proposition II, *g*, *h*, *&c.*) And even if they were debilitated, the part would not be distended by the vis a tergo (Proposition I.), and of course no increased flow could occur. The fluids are evidently attracted to the part irritated, and as dilatation must precede their entrance, I infer that such dilatation, excited by stimuli, is the immediate cause of all such local determinations of blood. This view is confirmed by the fact, that the afflux is proportioned to the irritability, and vascularity of the part and to the severity of the stimulus. They increase and decrease *pari passu.* The contraction of the heart, acting upon a single column of blood, can have no effect in increasing or diminishing the flow of blood to any tissue. Such local changes depend therefore, on local causes, and chiefly on the varying degree of excitement in each part.

"The influence of the capillaries, in drawing blood from the larger vessels is often exemplified in the operation of sinapisms, blisters, and other rubefacients in prostrated states of the system,—where the surface is cold and pale or lived, where the pulse can hardly be felt, aud the phenomena of life are almost extinguished. What is the modus operandi of such remedies in exciting reaction? Do they first increase the vis a tergo, the actions of the heart and arteries, that the capillaries may be distended? or do they not primarily excite the torpid capillaries, whence dilatation and afflux of fluids, and afterwards secondarily and sympathetically, the arteries and heart? The last view seems correct, because such local irritations are often excited, when the arteries and heart do not sympathize; and in all cases, the capillary excitement precedes the general excitation.—pp. 27—29.

"The rapid enlargement of the capillary and smaller arteries after the main artery of a limb has been tied was alluded to in Proposition II. *l.* The afflux of fluids to the cold and bloodless limb is no less evident than the dilatation of the vessels, and as the vis a tergo is diminished, while the redness and temperature are augmented, we must infer that the blood was moved by some other power than that derived from the heart and arteries; that the excited capillaries absorbed blood from the superior portions of the limb.

Dr. N. R. Smith, of the University of Maryland, records an interesting example of this kind*, in which a ligature was applied to the anterior tibial artery for a wound of this vessel. The hœmorrhage from the wound, and the pulsation of the artery on the top of the foot, ceased on drawing the ligature; but after some hours, the bleeding returned from the inferior orifice. The artery on the in-

*Philadelphia Monthly Journal, vol. ii. p. 18.

step now pulsated vigorously, the beat was stronger and more full in the wounded limb than in the other, and the blood, on experiment, was found flowing in a retrograde direction. The circulation here was more rapid by anastomosing channels than when the blood pursued its direct course.

"Professor SMITH was called upon to amputate the leg below the knee for a dry gangrene of the foot and ancle. The great arteries were found ossified, being mere tubes of bone. "Though all pressure was removed from the artery above, and no other means were used to suppress the hœmorrhage, yet not half a table spoonful of blood followed the operation. At the same time, the action of the heart, and of the arteries at the wrist, was vigorous."—p. 205. As no coagula existed in the arteries, and as the anastomosing vessels were not enlarged while the parts below had been supplied with some blood, it results that blood must have passed through these ossified tubes before the operation, and that its motion was not owing to the vis a tergo, as the open ossified tubes did not bleed when divided. Hence "the active power lost by the division of the arteries, resided in the capillary system of blood vessels of the foot and leg."—pp. 30—31.

Having, with these and many other similar facts, rendered it highly probable, that the capillaries, by their alternate dilatation and contraction, aid the heart in propelling forward the blood, he proceeds to say that, in many cases, they are, alone, adequate to the circulation. For the support of this assertion, he refers to comparative anatomy, and cites the invertebral animals which have no heart, and still maintain a vascular circulation, and to fishes which have a single heart only, and that devoted to the branchiæ or gills, leaving to the vessels, themselves, the responsibility of maintaining the systemic circulation. In man, he notices the portal circulation, which can receive no aid from the heart, except through the capillaries of the abdominal viscera. We have no doubt, however, that the hepatic capillaries are aided by the suction power of the heart and chest, especially the latter, the influence of which must be greatest immediately without its walls and floor, consequently, cannot, any where, be more efficient, than in that portion of the vena cava ascendens, which receives the hepatic veins. Omitting any further reference to the proofs by which he has sustained this proposition, we shall extract his concluding remarks:

"From the facts and arguments which have been presented for the support of the present Proposition, it results that the contraction of the heart and arteries is not of itself adequate to the propulsion of the blood; and that capillaries, possessing a dilating power, draw blood into their cavities, and thus assist the impetus derived from the heart. The combined influences, derived from the contraction of the heart, arteries, and capillaries, impel the blood forward into the veins, where further assistance is rende ed by the contractile power of these vessels, and when near the heart, by the suction influence of this organ and of the thorax. All parts of the circulatory apparatus thus assist each other, instead of furnishing obstacles to the circulation of the blood, as maintained in the present systems of physiology.—p. 34.

Our author's fourth proposition, that, *during the activity of inflammation an increased quantity of blood flows thro' the affected part*, is sustained by a series of facts, which we regard as altogether conclusive. To our apprehension, indeed, all the phenomena of active inflammation indicate increased *transmission*, not less than increased *accumulation*. The scarlet hue, increased heat, augmented sensibility and size, turgescence of the veins leading from the part, when if, obstruction and stagnation existed in it, they should be comparatively empty, finally, the copious hœmorrhage which ensues from incisions, are signs, not to be misunderstood, of great activity, as well as great fullness of the vessels. In addition to facts of this kind, our author has cited many others which we must pass over, to present the deductions which he has made from them:

"The phenomena and consequences of inflammation thus evince the close analogy which exists between this morbid state and those various processes of the animal system, in which every one recognizes an increased circulation; such as the renewal of a stag's horns, the growth of tumors, the erection of the penis and nipple, the enlargement of the uterus, the distention of the mammæ, and the subsequent secretion of milk, &c. The one class of phenomena manifests as decidedly as the other all the evidences of capillary excitement, and of an increased flow of blood through the affected tissues–.p. 36.

Having travelled over the series of facts and observations which constitute the body of his essay, we shall finish our extracts, with his final conclusions:

"If the object of this essay has been attained, it results that expansibility is a vital property, as well as contractility, and that both are modifications of the irritability of many modern pathologists; that the heart, arteries and capillaries, possess this property of dilating under the influence of their normal stimuli, but do not enlarge from any vis a tergo to which they are subjected; and finally, that they contribute by active expansion and contraction, at regular or irregular intervals, to the ready transmission of that vital fluid which is essential, not only to secretion and nutrition, but also to reparation.—pp. 36—37.

In bringing our notice of Dr. Hodge's paper to a close, we feel it but a duty to say, that it has merits of no ordinary stamp. It is true, that his fundamental propositions are not original; but they were not established; and, although, he has brought to their support, but little not already known, he has done that which entitles him to much praise. He has clearly set forth the doctrines of which he is an advocate, and concentrated upon them a greater body of facts and arguments, than any other writer with whom we are acquainted. These have been industriously, drawn from every available source, are accurately expressed, and logically arranged. Every page bears the impress of an acute and disciplined mind; enriched with the learning requisite to the undertaking, and capable of that profound investigation, which the subject of the essay so imperiously requires.

To say that his dissertation contains nothing objectionable, would be to bestow upon it the praise, which could not, in sober truth, be awarded to any professional essay extant; but we deal in no exaggeration, when we pronounce it highly interesting, and recommend it to the attentive perusal of all our American brethren.

